# Increased risk of postoperative complications after delayed stoma reversal: a multicenter retrospective cohort study on patients undergoing anterior resection for rectal cancer

**DOI:** 10.1007/s00384-025-04831-y

**Published:** 2025-02-13

**Authors:** Eihab Munshi, Josefin Segelman, Peter Matthiessen, Jennifer Park, Martin Rutegård, Olle Sjöström, Henrik Jutesten, Marie-Louise Lydrup, Pamela Buchwald, the RectoLeak Study group

**Affiliations:** 1 Department of Surgery, Skåne University Hospital, Lund University, Malmö, Sweden; 2 Department of Surgery, University of Jeddah, Jeddah, Saudi Arabia; 3 Department of Molecular Medicine and Surgery, Karolinska Institutet, and Department of Surgery, Ersta Hospital, Stockholm, Sweden; 4 Department of Surgery, Faculty of Medicine and Health, Örebro University, Örebro, Sweden; 5 Department of Surgery, Scandinavian Surgical Outcomes Research Group (SSORG), Institute of Clinical Sciences, Sahlgrenska Academy, University of Gothenburg, Gothenburg, Sweden; 6 Department of Diagnostics and Intervention, Surgery, Umeå University, Umeå, Sweden

**Keywords:** Defunctioning stoma, Stoma reversal time, Anterior resection, Anastomotic leak, Postoperative complications, Stoma reversal complications

## Abstract

**Purpose:**

Defunctioning stoma (DS) has been suggested to mitigate the consequences of anastomotic leak (AL) after low anterior resection. Stoma reversal (SR) is commonly delayed for nonmedical reasons in many healthcare systems. This study investigated the impact of the elapsed time from AR to SR on postoperative 90-day complications. The secondary aim was to explore the independent factors associated with a delayed SR.

**M&M:**

This multicenter retrospective cohort study included rectal cancer patients who underwent anterior resection (AR) and DS between 2014 and 2018. Multivariable logistic regression was used to evaluate the influence of the elapsed time from AR to SR on postoperative complications within 90 days.

**Results:**

Out of 905 patients subjected to AR with DS, 116 (18%) patients experienced at least one postoperative 90-day complication after SR. Multivariable analysis revealed an association between the elapsed time to SR and complications within 90 days from SR (OR 1.02; 95% CI, 1.00–1.04). The association with SR complications was further highlighted in patients who experienced delayed SR > 6 months after AR (OR 1.73; 95% CI, 1.04–2.86). AL after AR and nodal disease were both related to delayed SR.

**Conclusion:**

This study demonstrated that postoperative 90-day complications are associated with the time elapsed to SR. These findings emphasize the importance of early SR, preferably within 6 months, to prevent complications.

**Supplementary Information:**

The online version contains supplementary material available at 10.1007/s00384-025-04831-y.

## Introduction

After sphincter-preserving surgery, a defunctioning stoma (DS) temporarily diverts the fecal stream. Typically, DS is a defunctioning loop ileostomy. A DS mitigates the severity of clinically relevant anastomotic leak (AL) and reduces the incidence of reoperation for AL [1–4]. However, the DS has several drawbacks [5]. Early defunctioning loop ileostomy morbidity includes high-flow dehydration and renal injury [6, 7]. The long-term presence of DS may lead to stoma prolapse, parastomal hernia, and reduced anorectal function, often known as low anterior resection syndrome (LARS) [8, 9]. These stoma-related morbidities can result in increased costs and hospitalization [10, 11]. Moreover, one of the four defunctioning loop ileostomy procedures may become permanent because patient frailty, tumor progression, or pelvic inflammation following AL hinder the restoration of bowel continuity, resulting in the need for permanent maintenance or conversion to end colostomy [12, 13].

Since symptomatic AL most often manifests within 2 weeks following surgery, while stoma-related complications may occur continuously until stoma reversal (SR), experts recommend the reversal of DS between 8 and 12 weeks following the initial surgery [6, 14]. Previous studies have indicated that the reversal of a DS after 6 months may lead to a higher complication rate and longer hospital stays and nearly quadruples the risk of long-term bowel dysfunction [15, 16]. Stoma reversal is generally straightforward but has a 3–40% complication rate [17]. Despite its role as the second step in oncological surgery, approximately one-third of SRs are delayed due to low priority [11, 18].

The primary objective of this study was to investigate the relationship between the elapsed time to SR and postoperative 90-day complications post-SR. The secondary aim was to explore the independent factors associated with a delayed SR. We hypothesize that postponing SR will increase the likelihood of postoperative 90-day complications.

## Materials and methods

This project stems from the RectoLeak project, which involved eleven hospitals from Sweden, consisting of patients subjected to anterior resection (AR) for rectal cancer in the years 2014–2018, described in detail elsewhere [19]. Patients were identified from hospital operation lists, and data were collected via the Swedish ColoRectal Cancer Registry (SCRCR) and medical chart review. The SCRCR comprises nearly all rectal cancer patients (98.8%) in Sweden and has high validity [20]. This study was approved by the Swedish Ethical Review Authority (Dnr 2020–02285).

Following the planned AR, patients were followed up at outpatient clinics for symptoms and signs of adverse surgical events, including AL. Anastomotic leak after AR was defined as a defect in the intestinal wall at the site of the anastomosis (where the staple or suture line is located) that results in communication between the intraluminal and extraluminal compartments, which was proposed by the International Study Group of Rectal Cancer (ISREC) [21]. Patients without any signs of AL at endoscopic or radiological contrast examinations were scheduled for SR if not planned for adjuvant chemotherapy. Patients indicated for adjuvant chemotherapy received treatment before SR. Stomas that reversed over 6 months after the index surgery were classified as delayed SR. This specific cutoff time was related to increased postoperative complications after SR [15, 22]. The stoma status was followed for at least 2 years. Following SR, medical charts were reviewed for medical and surgical complications, including reoperations, within 90 days.

SR complications included mechanical small-bowel obstruction, ileus (paralytic ileus lasting more than 7 days or requiring total parenteral nutrition), bleeding, surgical-site infection (SSI), AL, wound dehiscence, incisional hernia, cardiac (new onset arrhythmia, myocardial infarction, and heart failure), and pulmonary events (pneumonia, respiratory aspiration, and acute respiratory distress syndrome). The type of reoperation after SR and the indications for the procedures were recorded.

### Data analysis

The correlation of the elapsed time from AR to SR (exposure) with postoperative 90-day complications after SR (outcome) was assessed. Elapsed time was defined as a continuous variable (in months) as well as a binary variable (> 6 months, i.e., delayed SR). The outcome was specified as SR complications or reoperations within 90 days from the time of SR.

To ensure a more consistent group for analysis, we excluded patients with AL from the primary outcome evaluation. This decision was impacted by the potential risk associated with Grade C AL, which can lead to the closure of the diverting stoma while performing an end colostomy [21].

However, while evaluating delayed SR-associated clinical factors, we investigated factors that are clinically relevant for determining the timing of SR, including age, sex, American Society of Anesthesiologists (ASA) physical score, AL grade, and TNM stage, in the entire cohort.

### Statistical methods

Categorical variables were compared via the chi-square test and Fisher’s exact test and are presented as percentages. The Mann‒Whitney *U* test was used to analyze continuous variables, expressed as medians with interquartile ranges (IQRs).

Logistic regression was used to analyze the correlation between the elapsed time to SR and the outcome (postoperative 90-day complications and reoperation after SR). Elapsed time was defined as a continuous variable (in months) and as a binary variable (> 6 months, i.e., delayed SR). The following factors were included in the multivariable logistic regression to adjust for confounding variables: age (≤ 65 and > 65), sex (female and male), ASA (I, II and III, IV), *yp/pT* stage (0, 1, 2 and 3, 4), and *yp/pN* stage (0 and 1, 2). The confounders were chosen based on clinical reasoning, suggesting an influence on the timing of the SR procedure and the occurrence of complications.

Univariable and multivariable analyses assessed the clinical factors related to delayed SR. A two-sided *p* value of < 5% was considered significant.

A complete case analysis was performed when data were missing, and > 2% of the missing data are reported in the tables. SPSS 28 software was used (SPSS, Chicago, IL, USA).

## Results

### Patient demographic and clinical characteristics

**Fig. 1:**
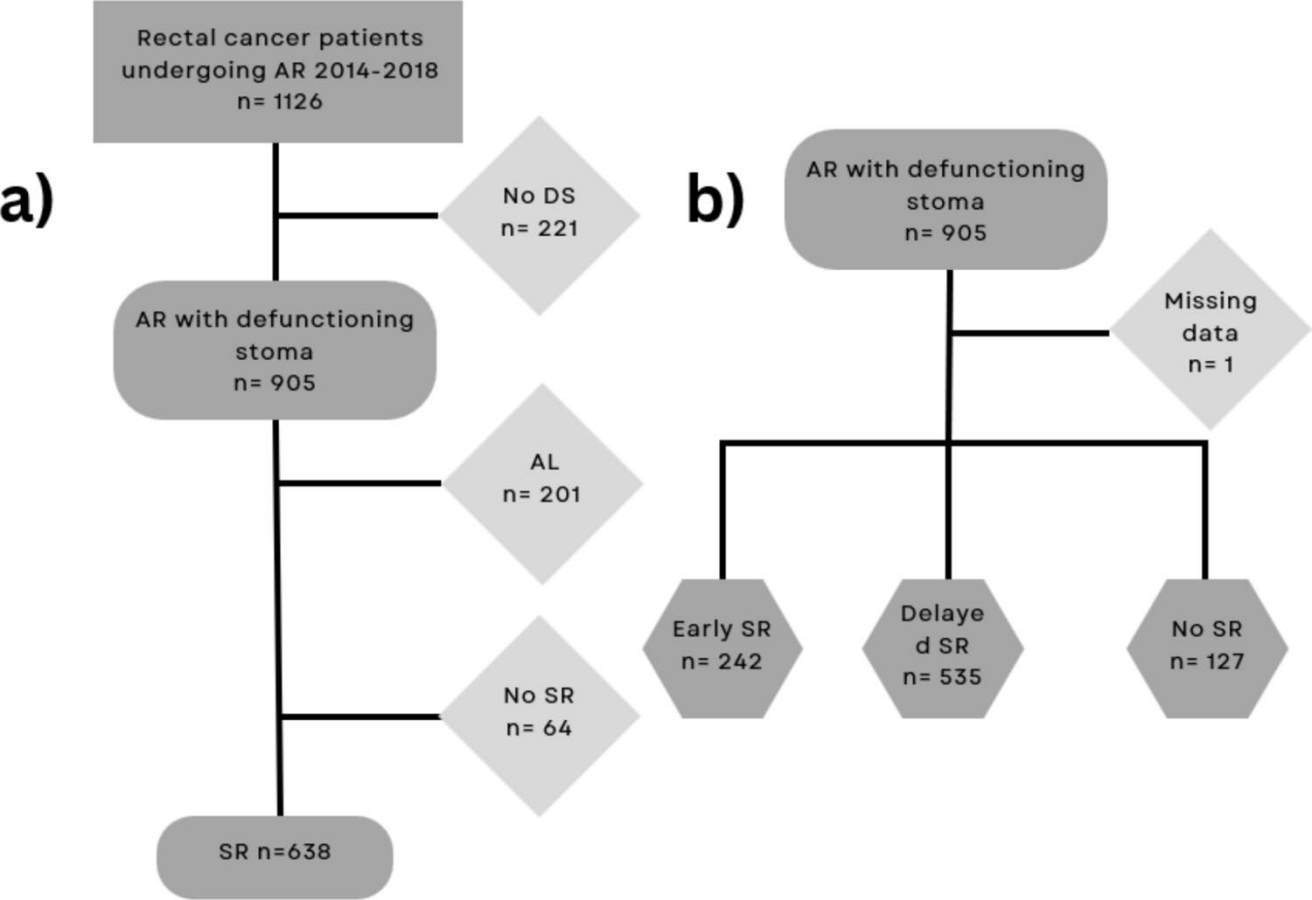
Flowcharts of patients who underwent anterior resection for rectal cancer between 2014 and 2018. **a** The elapsed time to stoma reversal and postoperative 90-day complications (primary endpoint) were studied. **b** Analysis of the clinical factors associated with delayed stoma reversal (secondary endpoint). AR, anterior resection; DS, defunctioning stoma; AL, anastomotic leak; SR, stoma reversal

Between 2014 and 2018, 11 hospitals in Sweden performed 1126 AR procedures for rectal cancer. The 905 patients who received a DS (898 defunctioning loop ileostomy, 99%) in conjunction with AR were included in the current study (Fig. 1). In total, 201 (22%) patients had AL. Table 1 displays the characteristics of patients without AL who had an SR or did not have one. Two hundred ninety-four (46%) patients underwent minimally invasive procedures, and 186 (29%) underwent robotic-assisted operations. After a mean follow-up of 4.1 years, 64 (9%) patients retained their DS. In the non-SR group, patients were older, more frequently classified as ASA III–IV, had more advanced tumors in terms of T and N stages, and a greater proportion had distant recurrence. In the SR group, half of the patients received neoadjuvant therapy—either radiotherapy, 216 (34%), or radiochemotherapy, 108 (17%)—while one-third received adjuvant chemotherapy, 217 (34%). Those who underwent adjuvant chemotherapy had a longer elapsed time to SR than those in the non-adjuvant group (11.4 vs. 6.4 months). Supplementary Table 1 compares the characteristics of the entire study group, including patients with AL.

**Table 1:** Clinical characteristics of stoma reversal compared with the non-reversed stoma, where patients with anastomotic leak are excluded

	Stoma reversal ***N* = 638**	Non-reversed stoma ***N* = 64**
**Age** in years (IQR)	66 (59–72)	70 (64–74)
**Sex** (male)	391 (61.3)	33 (51.6)
**BMI** (kg/m^2^) (IQR)	25.4 (23.2–28.1)	25.6 (23.4–28.4)
**Smoking**	41 (6.8)	4 (7.4)
Missing	33 (5.2)	10 (15.6)
**ASA class**		
I	146 (23.1)	7 (10.9)
II	391 (61.9)	40 (62.5)
III–IV	95 (15)	17 (26.6)
**CCI group**		
0	437 (68.5)	38 (59.4)
1	116 (18.2)	15 (23.4)
2	56 (8.8)	5 (7.8)
3 +	29 (4.5)	6 (9.4)
Tumor distance*		
High (11–15)	246 (38.6)	27 (42.2)
Middle (6–10)	378 (59.3)	36 (56.3)
Low ≤ 5	13 (2)	1 (1.6)
**Neoadjuvant therapy**		
Radiotherapy	216 (33.9)	23 (35.9)
RCT	108 (16.9)	15 (23.4)
None	314 (49.2)	26 (40.6)
**pCR**	9 (1.4)	3 (4.7)
**Operation time, minutes (IQR)**	272 (200–362)	263 (197–352)
**Surgical approach**		
Open/converted	343 (53.8)	42 (65.6)
Laparoscopy	108 (17)	10 (15.6)
Robotic	186 (29.2)	12 (18.8)
**Anastomosis type**		
End-end	155 (24.3)	14 (21.9)
Side-end/J-pouch	453 (71)	44 (68.8)
Missing	30 (4.7)	6 (9.4)
**Mesorectal excision type**		
TME	566 (88.7)	57 (89.1)
PME	72 (11.3)	7 (10.9)
**Intraoperative complications**		
Significant bleeding > 500 ml	66 (10.3)	8 (12.5)
Serosal tear	11 (1.7)	2 (3.1)
Bowel perforation	10 (1.6)	2 (3.1)
Ureteric injury	2 (0.3)	0
**Tumor stage**		
*yp/pT*		
T0	12 (1.9)	4 (6.3)
T1-2	261 (41.5)	21 (32.8)
T3-4	353 (56)	39 (60.9)
Tx	4 (0.6)	0
*yp/pN*		
N0	401 (63.8)	28 (43.8)
N1-2	226 (35.9)	36 (56.3)
Nx	2 (0.3)	0
M		
M0	603 (94.5)	58 (92.1)
M1	19 (3)	5 (7.9)
**Adjuvant chemotherapy**	217 (34)	28 (43.8)
Local recurrence	12 (1.9)	1 (1.6)
Distant recurrence	99 (15.5)	18 (28.1)
Follow-up (years)	4.8 (3.6–6)	4.6 (3.5–5.8)
**Stoma reversal time**		
≤ 6 months	209 (32.8)	NA
> 6 months	428 (67)	NA

Values in parentheses are percentages if not stated otherwise

*BMI* body mass index, *ASA* American Society of Anesthesiologists, *CCI* Charlson Comorbidity Index, *RCT* radiochemotherapy, *pCR* complete pathological response, *MIS* minimally invasive surgery, *IMA* inferior mesenteric artery, *ICG* indocyanine green, *TME* total mesorectal excision, *PME* partial mesorectal excision, *yp/pT* neoadjuvant/pathological tumor stage, *yp/pN* neoadjuvant/pathological lymph node stage, *NA* not applicable

*cm from the anal verge

^1^Mann-Whitney *U*

^2^Fisher’s exact test

^3^Chi-squared test

### Elapsed time to stoma reversal and postoperative 90-day complications

After patients who had AL (*n* = 201 (22%)) or did not undergo SR (*n* = 64 (7%)) were excluded, 638 remained in the main analysis regarding time to SR. One hundred sixteen (18%) patients experienced at least one postoperative 90-day complication after SR, and 39 (6%) patients underwent reoperation after SR (Suppl Table 2). The median time from AR to SR was longer in the postoperative 90-day complications group than in the non-complication group (10.2 (IQR 6.5–15.2) months vs. 7.3 (IQR 4.8–11.7) months (*p* < 0.001)). Similarly, the median time to SR was longer in the reoperation after SR group than in the non-reoperation group (11.1 (IQR 7.8–15) vs. 7.6 (IQR 4.9–12) months (*p* = 0.002)).

Patients were allocated to two groups: 209 (33%) patients had SR within 6 months of AR, and 428 (67%) patients had delayed SR (> 6 months). The univariable analysis revealed a relationship between delayed SR and postoperative 90-day complications and reoperation after SR (*p* = 0.009 and *p* = 0.021, respectively).

Supplementary Table 2 details the indications for and types of reoperations and overall complications.

The results of the multivariable analyses exploring the associations between time (continuous variable) from AR to SR and the main outcome of 90-day complications, as well as reoperations following SR, are presented in Table 2. After adjustment for clinical and pathological factors, a significant association was found between time and postoperative 90-day complications (OR 1.02 (95% CI, 1.00–1.04, *p* = 0.049)) but not with reoperations. In addition, delayed SR (SR > 6 months) was associated with an increased risk of postoperative 90-day complications (OR 1.73 (95% CI, 1.04–2.86, *p* = 0.034) (Table 3)) as well as reoperation after SR (OR 2.66 (95% CI, 1.06–6.68, *p* = 0.038)).

**Table 2:** Logistic regression relating elapsed time (per month) from AR to stoma reversal and overall postoperative complications and reoperation post-SR

**Overall postoperative complications**			
Unadjusted		Adjusted*	
OR (95% CI)	*p*	OR (95% CI)	*p*
1.02 (1.00–1.04)	**0.023**	1.02 (1.00–1.04)	**0.049**
**Reoperation**			
Unadjusted		Adjusted*	
OR (95% CI)	*p*	OR (95% CI)	*p*
1.01 (0.99–1.04)	0.346	1.01 (0.98–1.04)	0.510

*Adjusted for age, sex, ASA, T stage, and N stage

**Table 3:** Logistic regression relating elapsed time (> 6 months) from AR to stoma reversal and overall postoperative complications and reoperation post-SR

**Overall postoperative complications**			
Unadjusted		Adjusted*	
OR (95% CI)	*p*	OR (95% CI)	*p*
1.86 (1.17–2.97)	**0.009**	1.73 (1.04–2.86)	**0.034**
**Reoperation**			
Unadjusted		Adjusted*	
OR (95% CI)	*p*	OR (95% CI)	*p*
2.96 (1.22–7.17)	**0.017**	2.66 (1.06–6.68)	**0.038**

*Adjusted for age, sex, ASA, T stage, and N stage

### Clinical factors related to delayed defunctioning stoma reversal

Table 4 presents the uni- and multivariable analyses of the potential associations between clinical factors and a delayed SR (SR > 6 months). The analyses identified AL and N1-2 disease as independent factors related to delayed SR. No discernible influence was observed for age, sex, ASA class, AL grade, or T or M stage.

**Table 4:** Logistic regression was used to analyze patients’ clinical factors related to delayed stoma reversal (> 6 months from anterior resection)

Variables	Univariable analysis			Multivariable analysis		
	OR	95% CI	*p* value	OR	95% CI	*p* value
Age > 65	1.07	0.79–1.45	0.696	0.99	0.72–1.39	0.956
Sex (male)	1.34	0.98–1.83	0.075	1.36	0.96–1.91	0.082
ASA class (III–V)	1.26	0.81–1.96	0.329	1.12	0.70–1.81	0.629
Anastomotic leak	**2.11**	**1.41–3.53**	** < 0.001**	**2.30**	**1.41–3.75**	** < 0.001**
AL grade (B-C)	0.93	0.32–2.73	1			
Tumor stage						
yp/pT (T3-4)	**1.86**	**1.36–2.54**	** < 0.001**	1.21	0.86–1.71	0.272
yp/pN (N1-2)	**4.15**	**2.83–6.23**	** < 0.001**	**4.15**	**2.73–6.31**	** < 0.001**
M	2.03	0.68–6.05	0.245			

*ASA* American Society of Anesthesiologists physical status classification, *AL* anastomotic leak, *yp/pT* neoadjuvant/pathological tumor stage, *yp/pN* neoadjuvant/pathological lymph node stage

## Discussion

This study demonstrates an association between the time elapsed from AR to SR and postoperative 90-day complications after SR. Furthermore, patients with delayed SR (> 6 months) had a 1.7-fold increased likelihood of postoperative 90-day complications and a 2.7-fold increased likelihood of reoperation after SR. Nodal disease and AL post-AR impacted the timing of SR and were associated with its delay. For nodal disease, adjuvant chemotherapy is recommended, which may delay SR. When managing AL complications, an extended period for management and recovery is often needed before SR can be considered. Although the SR procedure is relatively straightforward, the SR timing could be crucial for obtaining preferable results.

Recently, Hacim et al. reported similar results in a cohort of 133 patients with defunctioning loop ileostomy, where the time until SR was classified into < 3, 3–6, and > 6 months [22]. The time to SR was found to be the primary determinant of complications related to defunctioning loop ileostomy, with complication rates of up to 62.5% in the latter group. These findings were consistent following stratification for complication type (ileostomy site-related, anastomosis-related, and metabolic) and severity [22]. Other prior studies have indicated that complication rates following SR vary between 13 and 40%, and the elapsed time to SR has been suggested as an independent risk factor for complications. Specifically, any SR delay of 6 months or more puts the patient at risk of experiencing complications [17, 23, 24]. A large retrospective cohort study reported a 6% increase in post-SR complications per month of delay [25]. However, some studies have reported that delaying SR has no effect on postoperative complications but have reported increased risks of postoperative complications among patients over 80 years of age, males, and those who experienced surgical site infection following AR [26, 27]. The conflicting results may be attributed to the studies’ limited reporting of 30-day follow-up after SR, possibly underestimating the influence of elapsed time.

A systemic review and meta-analysis investigated early SR (within 2 weeks post-AR) and concluded it is safe and feasible. Despite not increasing the incidence of AL or overall complications, early SR is still associated with a higher incidence of significant complications and reoperation [18]. Early SR accounts for barely more than 2% of the SR patients in our cohort and is outside the scope of the current study.

Patients with DS who require adjuvant chemotherapy (one-third of our cohort) face a complex decision of whether to prioritize the initiation of adjuvant chemotherapy over SR. The effectiveness of adjuvant therapy diminishes when it is initiated after 8 weeks [28]. The presence of DS during chemotherapy exacerbates chemotherapy-induced diarrhea and gastrointestinal toxicity, potentially affecting treatment completion, dosing, and subsequently disease-free survival [29]. In our study, the non-SR group received more adjuvant chemotherapy (44% vs. 34% in the SR group). Therefore, novel treatment approaches with neoadjuvant chemotherapy may be beneficial in terms of timely SR [30]. Nationwide research by Hoshino et al. revealed no difference in total adjuvant chemotherapy doses between patients with and without DS but did find a dose reduction in DS patients and patients with early SR (≤ 6 months after LAR) compared with those with late SR (7–12 months) [31]. A meta-analysis examining the SR during or after adjuvant chemotherapy revealed no significant differences in overall perioperative complications, AL, SSI, ileus, or length of hospital stay between the two groups [32]. However, these studies did not explore the effect of SR on chemotherapy completion or dosage modification. Presently, the results of the German CoCStom study are awaited, assessing adjuvant chemotherapy completion early (8–10 days following tumor resection and before commencing adjuvant) vs. delayed SR (26 weeks after tumor resection and adjuvant therapy completion) [33, 34].

### Strengths and limitations

The study’s limitations include its retrospective design, which introduces a risk of selection bias. In some patients, SR may be intentionally delayed for unregistered reasons, such as a need for a longer recovery time from AR and chemotherapy or poor nutritional status, making earlier SR disadvantageous in terms of risk of postoperative complications. Residual confounding may be present due to unknown predictors or missing information regarding the ex-anastomotic technique (stapled or handsewn) or if laparotomy is performed. The complications were not classified via a grading system, such as the Clavien-Dindo score, which may have hampered comparisons across studies. To create homogeneous comparable groups, patients with AL of all grades were excluded since a Grade C AL can mandate early closure of the DS and the creation of an end colostomy. On the other hand, this restriction may have introduced a selection of patients with a lower risk of surgical complications in general, underestimating the actual risk of complications to SR. Furthermore, the time interval between AR and adjuvant chemotherapy and the completion of adjuvant chemotherapy and the SR were not recorded. Nevertheless, the study benefits from an adequate sample size, encompassing data from 11 centers (county as well as university hospitals), enhancing its representativeness and generalizability.

## Conclusion

While timely SR is advocated to alleviate patient suffering, our findings hold relevance in the context of today’s overstretched healthcare systems. This study highlights the importance of seeking early SR to minimize the risk of postoperative 90-day complications.

## Supplementary Information

Below is the link to the electronic supplementary material.

Supplementary file1 (DOCX 21 KB)

## Data Availability

The data supporting this study's findings are available from Svenska Kolorektalcancerregistret (SCRCR). Nevertheless, restrictions apply to the availability of these data, which were used under license for the current study and are not publicly available. Data are, however, available from the authors upon reasonable request and with the permission of SCRCR. SCRCR can be contacted via rccnorr.statistiker@regionvasterbotten.se.
