# Supplementary material for: Increased risk of postoperative complications after delayed stoma reversal: a multicenter retrospective cohort study on patients undergoing anterior resection for rectal cancer
Source: Int J Colorectal Dis. 2025 Feb 13;40(1):36. doi: 10.1007/s00384-025-04831-y (PMC11821667; doi:10.1007/s00384-025-04831-y)
Supplement: Supplementary file 1 — Supplementary file1 (DOCX 21 KB) [file 384_2025_4831_MOESM1_ESM.docx]

### Supplementary Table 1. Clinical characteristics of the stoma reversal and non-reversed stoma groups (entire cohort, including patients with AL).

|  | Stoma reversal  **N=778** | Non-reversal stoma  **N=127** |
| --- | --- | --- |
| Age | 66 (59-72) | 69 (64-74) |
| Sex (Male) | 492 (63.2) | 78 (61.4) |
| BMI | 25.5 (23.3-28.2) | 25.4 (23.7-28.7) |
| Smoking | 55 (7.5) | 8 (7.1) |
| **ASA class** |  |  |
| I | 184 (23.9) | 13 (10.3) |
| II | 467 (60.6) | 80 (63.5) |
| III-V | 119 (15.5) | 33 (26.2) |
| **CCI group** |  |  |
| 0 | 533 (68.5) | 71 (55.9) |
| 1 | 138 (17.7) | 31 (24.4) |
| 2 | 69 (8.9) | 13 (10.2) |
| 3+ | 38 (4.9) | 12 (9.4) |
| **Tumor distance*** |  |  |
| High (13-15) | 108 (14) | 20 (15.7) |
| Middle (7-12) | 621 (80.2) | 98 (77.2) |
| Low ≤ 6 | 45 (5.8) | 9 (7.1) |
| **Neoadjuvant therapy** |  |  |
| Radiotherapy | 279 (35.9) | 58 (45.7) |
| RCT | 137 (17.6) | 33 (26) |
| None | 362 (46.5) | 36 (28.3) |
| pCR | 13 (1.7) | 4 (3.1) |
| Operation time | 279 (206-369) | 254 (194-345) |
| **Surgical approach** |  |  |
| Open surgery | 359 (46.1) | 75 (59.1) |
| MIS, not converted | 360 (46.3) | 43 (33.9) |
| MIS, converted | 59 (7.6) | 9 (7.1) |
| **Anastomosis type** |  |  |
| End-end | 177 (22.8) | 28 (22) |
| Side-end or J-pouch | 569 (73.1) | 86 (67) |
| Missing | 32 (4.1) | 13 (10.2) |
| **Mesorectal excision type** |  |  |
| TME | 702 (90.2) | 117 (92.1) |
| PME | 76 (9.8) | 10 (7.9) |
| **Intraoperative complications** |  |  |
| Significant bleeding > 500 ml | 30 (3.9) | 6 (4.7) |
| Serosal tear | 14 (1.8) | 5 (3.9) |
| Bowel perforation | 15 (1.9) | 3 (2.4) |
| Ureteric injury | 2 (0.3) | 0 |
| Other | 8 (1) | 1 (0.8) |
| **Pathological tumor stage** |  |  |
| yp/pT |  |  |
| Tx | 4 (0.5) | 0 |
| T0 | 16 (2.1) | 5 (3.9) |
| T1-2 | 305 (39.2) | 43 (33.9) |
| T3-4 | 445 (57.2) | 78 (61.4) |
| yp/pN |  |  |
| Nx | 2 (0.3) | 0 |
| N0 | 493 (63.4) | 65 (51.2) |
| N1-2 | 274 (35.2) | 61 (48) |
| M |  |  |
| M0 | 739 (95) | 116 (91.3) |
| M1 | 22 (2.8) | 9 (7.1) |
| Adjuvant Chemotherapy | 259 (33.3) | 45 (35.4) |
| Local recurrence | 17 (2.2) | 4 (3.1) |
| Distant recurrence | 118 (15.2) | 41 (32.3) |
| Follow-up (years) | 4.8 (3.6-5.9) | 5 (3.4-6.1) |

The values in parentheses are percentages if not stated otherwise.

^1^Mann-Whitney U

^2^Fisher’s exact test

^3^Chi-square test

*cm from anal verge

*BMI*, body mass index; *ASA*, American Society of Anesthesiologists; *CCI*, Charlson Comorbidity Index; *RCT*, radiochemotherapy; pCR, complete pathological response; *MIS*, minimally invasive surgery; *IMA*, inferior mesenteric artery; *ICG*, indocyanine green; *TME*, total mesorectal excision; *PME*, partial mesorectal excision; *yp/pT*, neoadjuvant/pathological tumor stage; *yp/pN*, neoadjuvant/pathological lymph node stage.

### Supplementary Table 2. List of postoperative 90-day complications and reoperations following stoma reversal (SR)

| **Complications post SR** | **Stoma reversal**  **N= 638 (%)** |
| --- | --- |
| Cardiac | 3 (0.5) |
| Pulmonary | 4 (0.6) |
| SBO | 30 (4.7) |
| Ileus | 33 (5.2) |
| Bleeding | 2 (0.3) |
| SSI | 17 (2.7) |
| Infection (other than SSI) | 23 (3.6) |
| Leak | 14 (2.2) |
| Wound dehiscence | 4 (0.6) |
| Incisional Hernia | 10 (1.6) |
| Patients had a complication or more post-SR | 116 (18.2) |
| **Indication for reoperation post-SR** |  |
| SBO | 17 (2.7) |
| Bleeding | 4 (0.6) |
| SSI | 4 (0.6) |
| Wound dehiscence | 4 (0.6) |
| Small bowel AL | 6 (0.9) |
| New stoma creation | 8 (1.3) |
| Bowel resection | 9 (1.4) |
| Negative laparotomy | 7 (1.1) |
| Other | 4 (0.6) |
| Patients underwent reoperation post-SR | 36 (6,1) |

SBO; small bowel obstruction, SSI; surgical site infection, AL; anastomotic leak
